# Bactericidal and antioxidant properties of essential oils from the fruits *Dennettia tripetala* G. Baker

**DOI:** 10.1186/s12906-016-1459-4

**Published:** 2016-11-28

**Authors:** Sunday O. Okoh, Benson C. Iweriegbor, Omobola O. Okoh, Uchechukwu U. Nwodo, Anthony I.Okoh

**Affiliations:** 1 SAMRC Microbial Water Quality Monitoring Center, University of Fort Hare, Private mail Bag x1314, Alice 5700, Eastern Cape Province, South Africa; 2 Department of Biochemistry and Microbiology, University of Fort Hare, Private mail Bag x1314, Alice 5700, Eastern Cape Province, South Africa; 3 Department of Pure and Applied Chemistry, University of Fort Hare, Private mail Bag x1314, Alice 5700, Eastern Cape Province, South Africa

**Keywords:** *Dennettia tripetala*, Essential oils, Antibacterial, Antioxidant, Methyl phenyl formate

## Abstract

**Background:**

The antibacterial and antioxidant properties of the essential oils (EOs) of unripe and ripe fruits of *Dennettia tripetala* and their potential for the management of infectious and oxidative-stress diseases were investigated in-vitro in this study.

**Method:**

Essential oil obtained from the fruit in Clevenger modified apparatus, was characterized by high resolution GC-MS, while antioxidant and antibacterial properties were tested by spectrophotometric and agar diffusion methods respectively.

**Results:**

The EO demonstrated strong antibacterial properties when subjected to multi –drug resistant bacterial strains: *Enterococcus faecium* (ATCC19434), *Escherichia coli* (ATCC 700728), *Staphylococcus aureus* (NCINB 50080), *Listeria ivanovii* (ATCC 19119), *Enterobacter cloacae* (ATCC13047) and four previously confirmed multi resistant bacterial isolates from our laboratory stock culture. The unripe fruit oil (UFO) demonstrated greater activity than the ripe fruit oil (RFO) against most of the tested bacteria with minimum inhibition concentrations (MIC) ranging between 0.05–0.20 mg/mL while that of the ripe fruit oil (RFO) ranged from 0.10–0.20 mg/mL. The IC_50_ for RFO (0.62 ± 0.12 mg/mL) showed that it has higher antioxidant strength than UFO and vitamin C (0.87 ± 0.23 and 3.39 ± 0.12 mg/mL) but a lower activity compared to β-carotene (0.32 ± 0.22 mg/mL) in scavenging 2, 2-diphenyl-1-picrylhydrazyl radicals (DPPH^•^). The EOs also demonstrated strong ability in scavenging three other different radicals (ABTS, lipid peroxide and nitric oxide radicals) in concentration dependant -manner.

**Conclusion:**

Findings from this study suggest that apart from the local uses of the plant extracts, the EO has strong bioactive compounds, noteworthy antibacterial, antiradical properties and may be good candidates in the search for lead constituents for the synthesis of novel potent antibiotics.

## Background

Evidence from previous studies have indicated that essential oil (EO) is a potent antibacterial agent and as such could serve as an alternative source to combat certain pathogenic bacterial species, in addition to yeasts and filamentous fungi [1], as well as diseases associated with free radicals [2]. Components of EOs are numerous and a vast number have been shown to exhibit antibacterial functions, particularly; aromatic hydrocarbons, phenylpropenes, aliphatic and cyclic terpenoids [3]. Essential oils have been reported to rapidly diffuse bacterial cell membranes resulting in increased cell membrane permeability [4]. Consequently, leakage of vital intracellular constituents ensue [5] and ultimately, cell death occurs. Cell wall, cell membrane, intracellular proteins, enzymes and nucleic acids among others are important target sites for drug design and some essential oils constituents have been implicated to target these sites [6, 7].

Enzymatic endogenous antioxidants including catalase, superoxide dismutase, glutathione peroxidase attempt to rid oxidants in a physiological process, however, the constellation of radicals generated including lipid peroxyl (LP•), superoxide (O_2_•), nitric oxide (NO•), hydroxyl (HO•), consequent to metabolic activities and environmentally induced stress factors, overwhelms the naturally produced antioxidants. Cancers, arteriosclerosis, Alzheimer’s, asthma and arthritis, among others accrues with the cellular injury [8]. Plant decoctions, herbs, spices, infusions, and poultices now known to be rich in secondary metabolites have been used for years by man in the management of diseases before the written word. In recent years, phytochemical studies [5–9] have reported that secondary metabolites including alkaloids, flavonoids, and phenols from plants and their essential oils exhibit strong antioxidant activity. Essential oil may serve as a plausible alternative to synthetic antibiotics due its potential to diffuse microorganism cell membrane resulting in inhibition of cell growth as well as capacity to scavenge free radicals [10]. Essential oil constituents including carvacrol, carvone, caryophyllene, limonene and thymol have been reported to possess these properties [7, 11, 12].

*Dennettia tripetala* (pepper fruit tree) of the family *Annonaceae* is used as a spice and condiment in West Africa [13]. It is widely grown in the West African rainforest region including the south east and south west Nigeria. The matured *D. tripetala* fruit is green when unripe and yellow when ripe, both having a pungent and spicy taste. Previous studies on *D. tripetala* seed showed that it has antifungal and insecticidal attributes [13]. The leaf essential oil was reported by Oyemitan et al. [14] to possess significant antinociceptive and anti-inflammatory activities in rodents. Another study by Lewis and Ausubel [15] suggested that tannins, terpenoids and other phytochemicals in the plant could be responsible for wide the range of bioactivities of *D. tripetala.* The aqueous extract of the unripe fruit was demonstrated by Adebayo et al. [16] to possess better bioactivity than ripe fruit. However, there is dearth of information on the identities of ripe and unripe fruit essential oil constituents as well as comparative studies on antibacterial and antioxidant properties. This information is vital for comprehensive understanding and evaluation of the economic value of the plant. This current study aimed to evaluate the in-vitro antibacterial and antioxidant properties of the ripe and unripe fruit essential oil of *D. tripetala.*

## Methods

### Chemicals

The chemicals and reagents used included the following: Mueller Hinton agar from oxford Ltd (Hampshire, England), Dimethyl sulphur oxide (DMSO) and methanol from Fluka Chemicals (Buchs, Switzerland). 2, 2-azino-bis (3-ethylbenzothiazolin - 6-sulfonic acid) diammonium salt (ABTS), 2, 2-diphenyl-1-picrylhydrazyl (DPPH) were bought from Sigma - Aldrich St Louis, USA). Chemicals and reagents used were all analytical grade.

### Collection and processing of materials for study

Unripe and ripe fruits of *D. tripetala* were bought at the fruit and spice market, Mushin, Lagos, Nigeria. A plant taxonomist authenticated the plant and samples were kept in the Lagos University Herbarium (LUH) with voucher specimen number LUH7001A and LUH7001B for the ripe and unripe fruit respectively. The unripe and ripe fruits (150 g each) were separately milled and modified Clevenger apparatus was employed as previously described [17] to extract the essential oil. The hydrodistillation experiment was carried out thrice on the milled samples separately to obtain enough oil for bioactivity assays. The extracted EOs were dried in anhydrous sodium sulphate, left in tinted vials and stored at 4 °C. Essential oil yield (w/w %) of the fruit was then calculated.

### Characterization of essential oils by Gas chromatography-mass spectrometry (GC-MS)

GC-MS was utilized for analyzing and identifying the essential oil constituents. The GC-MS conditions were programmed as previously described [18], in which a Hewlett- Packed HP 5973 mass spectrometer interfaced with an HP 6890 gas chromatograph was used. Conditions of the temperature and column were; equilibration time 3.00 min, ramp 4 °C/min, initial temperature 70 °C, final temperature 240 °C; inlet: splitless, initial temperature 220 °C, pressure 8.27 psi, purge flow 30 mL/min, purge time 0.20 min, helium gas; column: capillary, 30 m × 0.25 mm, internal diameter 0.25 μm, film thickness 0.7 mL/min, average velocity 32 cm/sec; MS: EI method at 70 eV. Subsequently, identity of each constituent was carried out by agreement of their mass spectra data (MSD) with the reference held in the computer library (Wiley 275, New York). In addition, matching the retention index (RI) of each compound with those in literature was used in identifying the compounds. The peak areas were used to obtain total percentage composition of the essential oil.

## Antibacterial activity

### Bacteria suspensions test

Five multi-drug resistant reference strains of bacteria and four bacteria from our laboratory stock culture which have been confirmed as multi-resistant bacteria [19, 20] were used for the antibacterial test. The reference and laboratory strains included of four Gram positive: *S. aureus* (NCINB 50080), *E. faecium* (ATCC19434), *L. ivanovii, (*ATCC 19119), *E. cloacae* (ATCC 13047) and five Gram negative bacteria: *E. coli* O157 (ATCC 700728), *E. coli* 180, *E. coli* 179*, E. coli* 132 and *Vibro* spp. All bacterial strains which had previously been confirmed resistant to Ampicillin, Cefuroxime, Tetracycline, Nalidixic, Cephalexin, Sulphamethoxazole and Streptomycin [21] were tested against the essential oils and ciprofloxacin following CLSI (2014) guidelines. Both minimum inhibitory concentration (MIC) and minimum bactericidal concentration (MBC) potentials of the essential oils and controls were determined.

### Evaluation of the MIC and MBC

The micro dilution technique was carried out to evaluate the MICs. Eight hundred, 900, 950, 975 and 987.5 μL of Mueller-Hinton Broth (MHB) was dispensed into each Eppendorf tube. The essential oil stock extracted after evaporation of n-hexane was dissolved in DMSO (0.2 mL). Thereafter, aliquots of 200 μL, 100 μL, 50 μL, 25 μL and 12.5 μL were added respectively into each tube containing MHB to bring the final volume in each tube to 1 mL and the mixture was vortexed. The inoculum suspension (20 μL) of each test bacterial isolate (0.5 McFarland, ~1 × 10^8^ cfu/mL) was subsequently added and vortexed to permit adequate mixing of the essential oil and broth. Ciprofloxacin and DMSO were used as the positive and negative controls, respectively. This experiment was performed in duplicate and incubated at 37 °C for 24 h. The lowest concentration without visible growth was reported as the MIC.

The minimum bactericidal concentration (MBC) was tested by pour plate method of all tube content without visible growth in the MIC technique above onto fresh nutrient agar plates and the culture incubated for 24 h at 37 **°**C. The lowest concentration of extracts that did not yield any colony growth on the solid medium after the incubation period was regarded as minimum bactericidal concentration (MBC).

### Radical scavenging activity

Four different (DPPH, ABTS, nitric oxide and lipid peroxyl) radical scavenging tests were employed to study the antioxidant properties of *Dennettia tripetala* fruits volatile oil.

### DPPH test

The DPPH test on *D. tripetala* EO was performed as previously described by Liyana-Pathirana et al. [22] with slight modification. Briefly, a solution of DPPH (2.7 mM) in DMSO was prepared and 1 mL added to 1 mL of the essential oil dissolved in DMSO (0.05 - 0.50 mg/mL) as well as the reference compounds (RC). All solutions were then vortexed. The reaction mixtures were then incubated in the dark for 30 min at an ambient temperature. The absorbance of the reaction mixture was then read at 517 nm against a reference blank containing DMSO. The assay was carried out in triplicate. The essential oil’s potency to reduce DPPH^•^ to neutral molecule was computed as an inhibitory percentage using the following formula:

$$ \%\  inhibition = \left\{\left( Ab{s}_{control}\hbox{--} Ab{s}_{sample}\right)\right\}/\left( Ab s\  control\right)\ x\ 100 - ---------- - \left(*\right) $$

Abs _control_ is the absorbance of the DPPH radical + methanol; Abs _sample_ is the absorbance of DPPH radical + essential oil or reference compound (RC) and results expressed as means ± S.D. The regression equation generated from a standard curve was used to calculate the IC_50_ value of the extract as well as the RC, while *T*-Test analysis was employed to test significance difference of % inhibitions against the concentrations using SPSS15.0 for windows (IBM SPSS Inc *OLRAC SPS*). Significant difference was considered at a level of *P* < 0.05.

### ABTS test

The ABTS radical scavenging assay was carried out following the method of Re et al. [23] with some modification according to Witayapan et al. [1] by mixing 1:1 volumes of ABTS 7.0 mM and 4.9 mM potassium persulfate solution. The mixed solution was kept at room temperature for 12 h in a dark chamber. The ABTS radical cation (ABT^**.+**^) was then diluted with DMSO to equilibrate its absorbance to 0.705 (±0.001) at 734 nm. To carry out the assay, 1000 μL of the test samples in DMSO (0.05–0.50 mg/mL) were mixed with 1000 μL ABT^**.+**^ solution, bringing final volume of each mixture to 2 mL. The mixture was allowed to react for 7 min. The absorbance at 760 nm was measured spectrophotometrically. The radical scavenging activity of the EO or RC was expressed in term of percentage (%) inhibition of ABTS ^•+^ using expression in equation (*) described in DPPH assay. The assay was carried out in triplicate and average % inhibition calculated.

### Nitric oxide radical test

The nitric oxide radical scavenging activities of the essential oils were carried out according to the modified method described by Makhija et al. [24]. The compound sodium nitroprusside is known to decompose in aqueous solution at physiological pH (7.2) producing nitric oxide radicals (NO^**.**^). Under aerobic conditions, nitric oxide radicals react with oxygen to produce stable products (nitrate and nitrite) which can be measured using Griess reagent [25]. One milliliter of sodium nitroprusside solution (10 mM) was added to 1 mL of the essential oil at varying concentrations (0.05–0.5 mg/mL) and the mixture was then incubated at ambient temperature for 110 min. After incubation, 1 mL of the reacting mixture was added to Griess reagent (1%, sulphanilamide, 1% N-napthyl-ethylenediamine hydrochloride in 2% o- phosphoric acid). The absorbance of the colour developed was then measured at 546 nm against the reagent blank. The expression in equation (*) described in DPPH assay was used to obtain % of the scavenged nitric oxide radicals. The experiment was carried out thrice and average value calculated.

### Lipid peroxidation test

The inhibition of lipid peroxidation by the essential oils as measured using an adaptation of the method described by Badmus et al. [26] utilizing egg yolk as lipid rich media. The test samples (0. 1 mL) at varying concentrations (0.05 - 0.50 mg/mL) in DMSO was added 10% egg yolk homogenate (0.5 mL) and the reaction mixture made up to 1 mL. Lipid peroxidation was induced by addition of 0.05 mL of 0.07 M FeSO_4_ followed by a 30 min incubation at ambient temperature. Then, 1.5 mL of 10% acetic acid (pH 3.50) and 1.5 mL of 0.08% 2-thiobarturic acid in (1.1% sodium dodecyl sulphate and 20% trichloroacetic acid) were added and the mixture was vortexed and heated at 65 °C for one hour. Upon cooling, 0.5 mL of n-butanol was added to the reaction mixture and centrifuged for 10 min at 3000 rpm. The upper organic layer was then aspirated and the absorbance read at 532 nm. The percentage inhibition of lipid peroxidation was calculated using the expression in equation described in DPPH assay. The experiment was performed thrice and average % inhibition calculated.

### Cytotoxicity Test

The hemolytic method as described by Helander et al. [7] was used to test toxicity of the EOs, with some modifications. The in vitro hemolytic assay evaluates hemoglobin release in the plasma (as an indicator of red blood cell lysis) due to exposure to test agent. Blood agar plates were prepared using sheep red blood cells. The hemolytic activity of the essential oils were tested in wells bored on the plates at different concentrations (0.025 - 0.20 mg/mL) prepared in DMSO. Thereafter, the EO (30 μL) was added to each well and DMSO without the oil served as the negative control. The plates were incubated at 37 °C for 24 h. Thereafter, the wells were observed for the presence of hemolytic activity. This experiment was performed in duplicate.

### Analysis of data

Statistical analysis was performed using SPSS15.0 for windows (IBM SPSS Inc *OLRAC SPS* registration number 2012/1786646/07). All experimental results were expressed as means ± S.D of duplicate in the toxicity and antibacterial tests, while each antioxidant assay was carried out in triplicate. Percentage inhibition of radicals was concentration-dependent and linear regression equation generated from the standard curve for each antioxidant was used to calculate the IC_50_ value. *T*-Test correlation analysis was employed to test significant differences between the concentration and percentage inhibition. Significant difference was considered at a confidence level of *P* < 0.05.

## Results and discussion

### Chemical composition of Essential oils

The UFO as well as the RFO yields of *Dennettia. tripetala* was 0.62 and 1.10% respectively. Chemical composition of the EOs and identity of constituents are presented in Table 1. The chemical constituents of *D. tripetala* EOs which were predominantly terpenoids, effectively enhanced the antioxidant and antibacterial properties in the current research. Thirty three constituents were found in the RFO, while the UFO contained 27 compounds representing 95.32 and 94.06% of the total oil content respectively (Table 1). In the UFO, monoterpenoids accounted for 74.60% of the overall oil content and sesquiterpenoids content was 19.45%. The dominant monoterpenoids were 2-methyl phenyl formate (56.05%), α -terpinene (13.93%) and linalool (2.57%). Caryophyllene (6.23%), α –farsenesene (1.50%), *trans*-cadinol (0.58%), caryophyllene oxide (0.22%) and 1- (+) - ascorbic acid 2,6-dihexadecanoate (0.16%) were important the bioactive sesquiterpenoids identified in UFO. In the RFO, the monoterpenoids and sesquiterpenoids content increased to 75.10%, and 20.10% respectively. The quantity of some of the bioactive compound such as α -terpinene (16.25%), caryophyllene (10.80%), and linalool (3.85%) were significantly higher in ripe fruit. However, the monoterpenoid constituent (2-methyl phenyl formate) found in the RFO decreased to 51.26%.

**Table 1:** Components of volatile oils in unripe and ripe fruits of *Dennettia tripetala*

Component^a^	KI^b^	Composition (%)		Methodsof Identification	Mass spectra (MS) Data^c^	Q A^d^
		UFO RFO				
1-Nitro-pentane	900	0.31	0.68	MSD, RI	43,41,69,71	89
α-Pinene	927	0.05	0.10	MSD, RI	93,79, 41,136	99
β-Pinene	938	0.06	0.07	MSD, RI	93,69, 41, 136	98
Camphene	940	0.21	0.10	MSD, RI	93,69,41,77	99
β-Myrcene	944	0.20	0.40	MSD, RI	41, 93, 77,136	97
α- Phellandrene	953	–	0.20	MSD, RI	93,68,136,79	95
p-Cymene	957	0.13	0.30	MSD, RI	41,93,69,77	95
(+) - 4 - Carene	963	–	t	MSD, RI	145,41,135,128	90
β-Ocimene	968	0.08	0.27	MSD, RI	119,91,134,136	94
Linalool	970	2.57	3.85	MSD, RI	71,43,69,55	97
α -Terpinene	1031	13.93	16.25	MSD, RI	93,136,121,77	90
Phenylethyl alcohol	1045	0.61	0.38	MSD, RI	81,,69,55, 108	90
Ui	1048	0.39	0.30			37
Borneol	1116	t	–	MSD, RI	43,95,41,105	99
Terpinen-4-ol	1128	–	0.25	MSD, RI	71,93,111,41	96
α-Terpineol	1157	0.08	0.09	MSD, RI	71,93,111,41	99
Safrole	1278	–	0.60	MSD, RI	98,148,108	91
2-Methylphenyl formate	1356	56.05	51.26	MSD, RI	15, 106,77,51	89
Elemene	1385	–	0.06	MSD, RI	109, 43,95,161	98
Caryophyllene	1419	6.23	10.80	MSD, RI	41,93,133,79	99
Humulene	1459	0.54	0.36	MSD, RI	93,80,121,41	90
α -Farnesene	1472	1.50	0.74	MSD, RI	69,93,107, 133	95
β -Farnesene	1482	t	0.16	MSD, RI	67,107,93,133	93
Caryophyllene oxide	1579	0.22	0.70	MSD, RI	41,111, 79,93	94
Copaene	1633	0.09	0.04	MSD, RI	105,119,161,141	95
Ui	1678	–	1.59			45
4-epi-cubenol	1720	–	t	MSD, RI	43,105,119,161	90
Ui	1756	8.69	4.33			38
Guaiol	1785	0.10	0.14	MSD, RI	59,93,107,161	90
α-Eudesmol	1834	0.14	0.21	MSD, RI	77,51, 33, 29	89
*trans*-Cadinol	1872	0.58	0.63	MSD, RI	43,105,204,176	91
Azulen-5-ol	1895	0.07	0.09	MSD, RI	59,93,107,135	95
Ascorbic acid 2,6-dihexadecanoate	1904	0.16	0.07	MSD, RI	63,′156,174,118	98
9-Octadecenoic acid	1925	0.30	0.24	MSD, RI	209,253,344,44	93
Total oil content (%)		94.06	95.32			
Yield (% w/w)		0.62	1.10			

*Ui* Unidentified, *MSD* mass spectra data, *RI* retention index relative to C_9_- C_23_ on the column HB-5, t = less than 0.05%

^a^Components elution order in column HB-5;^b^ = Kovat’s index,^c^ = some of the m/z for most abundant peaks in the mass spectrum,^d^ = % of library quality assurance

The presence of phellandrene, (+) -4-carene, terpinen-4-ol, safrol and elemene was significantly higher in the ripe fruit oil, compared to the unripe fruit oil. This may indicate possible physiological effects of ripening which explains pigment change of *D.tripetala* fruit as reported previously by Adebayo et al. [16]. Linalool, caryophyllene, 4-carene, phenyl ethyl alcohol and 9-octadecenoic acid found in this present study were among the compounds reported by Elekwa et al. [27] as one the major compounds in the seed of *D. tripetala*. However, some of the reported bioactive compounds found in the RFO such as caryophyllene oxide [28], terpinen-4-ol [29], farnesene [30], and ascorbic acid [31] were not detected in the seed EO report of Elekwa et al. [27]. The study of Kumar et al. [32] revealed palmitic acid, eicosanoic acid, ethyl ester and linoleic acid as major components of *D. tripetala* solvent extracts*.* The discrepancy in the composition of *D. tripetala* essential oil grown in different regions in Nigeria and elsewhere may be due to differences in factors, such as climate, season, geographical conditions, age of the plant, humidity of the harvested plant material, extraction technique and the existence of chemotypes [33]. 1-nitro-2-phenylethane isolated by Oyemitan et al. [14] from dried seed essential oil of *D. tripetala* was not found in our EOs. However, the yield of 1-nitro-pentane (0.68%) identified in EOs of this study is higher than 1-nitro-2-phenylethane (0.59%) obtained from the seed EO of *D. tripetala* by Elekwa et al. [27]. This suggests the possibility of chemical transformation of 1-nitro-pentane in the seed EO. To the best of our knowledge the dominant compound 2-methyl phenyl formate found in this present study in *D. tripetala* EOs has not been reported as component of the plant. Methyl phenyl formate which naturally occurs in capsicum, coffee, pepper and some wine [34], may account for peppery characteristics when the unripe or ripe fruit of *D. tripetala* is consumed. The two EOs showed some hemolytic activity on Sheep red blood cells. This might be due to the fragile nature of the red blood cells since there has never been any report on human toxicity after consumption of *D. tripetala* fruits as it is a commonly consumed fruit in West Africa.

**Fig. 1:**
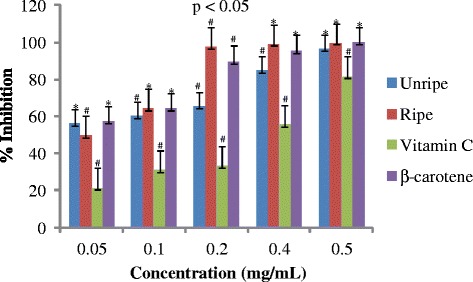
Antioxidant of fruit volatile oil in D. tripetala and reference compounds on DPPH radicals. * not significantly different, # significantly different (*p*< 0.05)

**Fig. 2:**
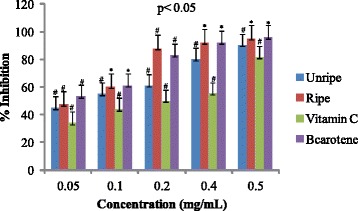
Antioxidant effects of fruit volatile oil in *D. tripetala* and reference compounds on ABTS radicals: # significantly different, * not significantly different (*p*< 0.05)

**Fig. 3:**
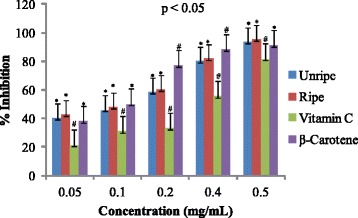
Antioxidant effects of fruit volatile oil in *D. tripetala* and reference compounds on lipid peroxidation radicals: * not significantly different, # significantly different (*p*< 0.05)

**Fig. 4:**
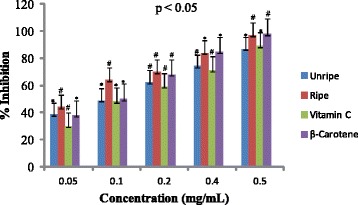
Antiradical activities of fruit volatile oil in *D. tripetala* and reference compounds on Nitric oxide radicals: * not significantly different, # significantly different (*p*< 0.05)

**Fig. 5:**
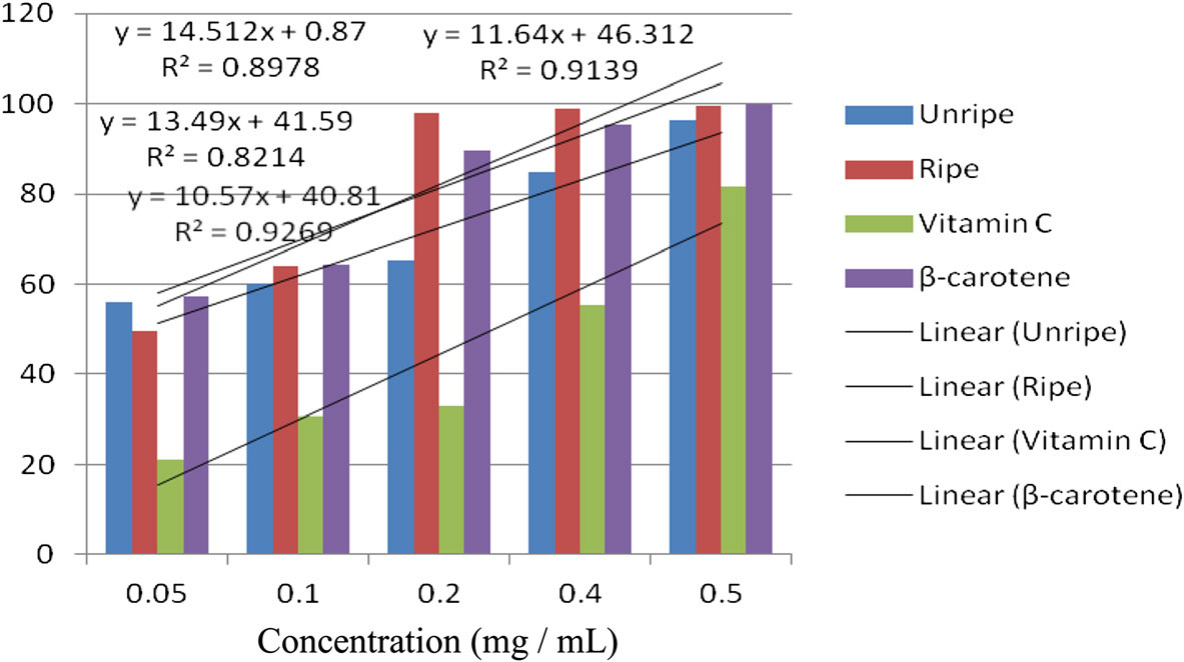
Antioxidant effects of fruit volatile oil in *D. tripetala* and reference compounds on radicals of DPPH (standard curves for the oils and ref compounds)

**Fig. 6:**
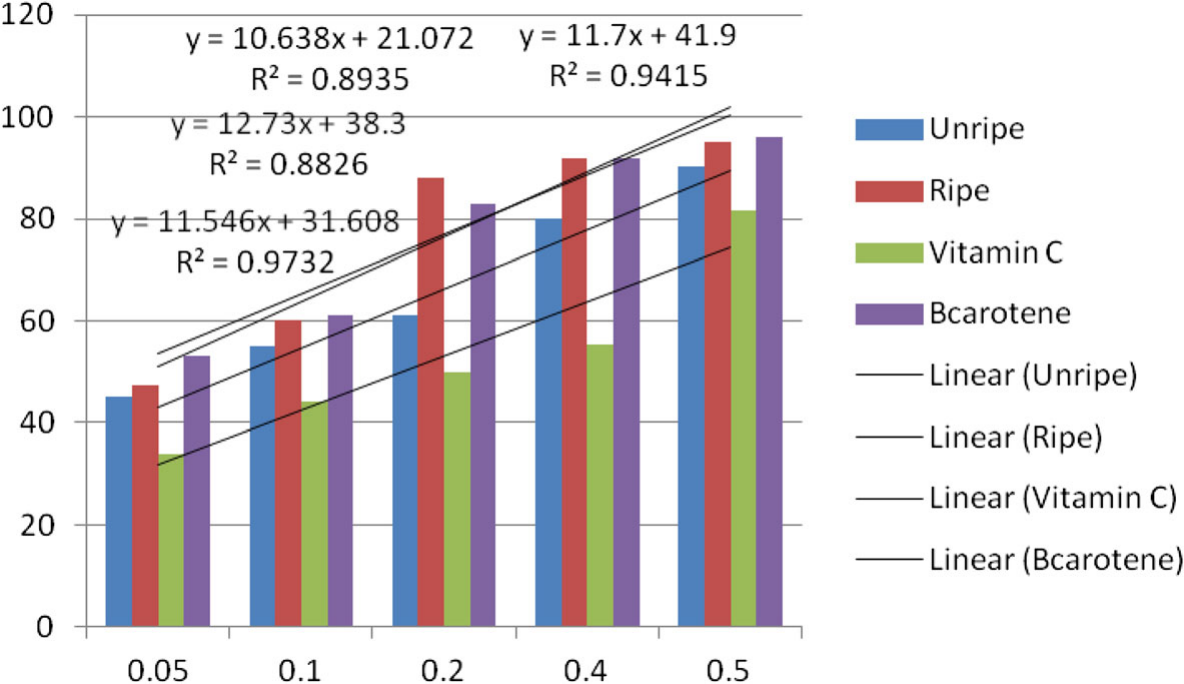
Antioxidant effects of fruit volatile oil in *D. tripetala* and reference compounds on radicals of ABTS (standard curves for the oils and ref compounds)

**Fig. 7:**
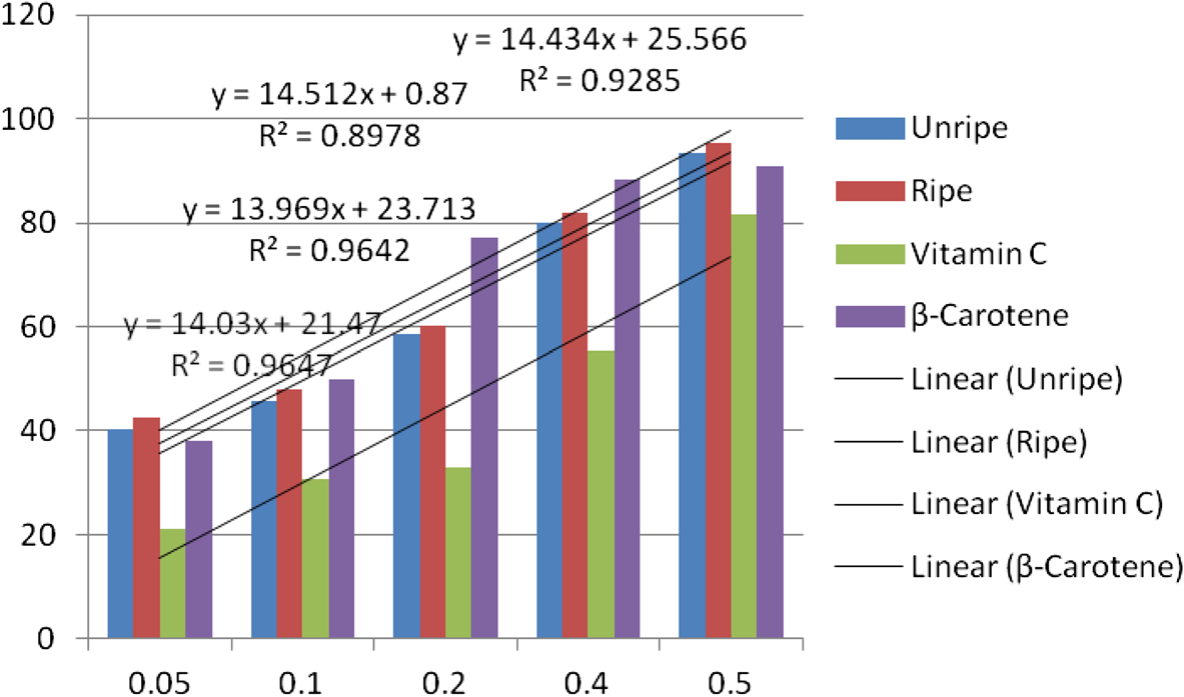
Antioxidant effects of fruit volatile oil in *D. tripetala* and reference compounds on radicals of lipid peroxidation (standard curves for the oils and reference compounds)

**Fig. 8:**
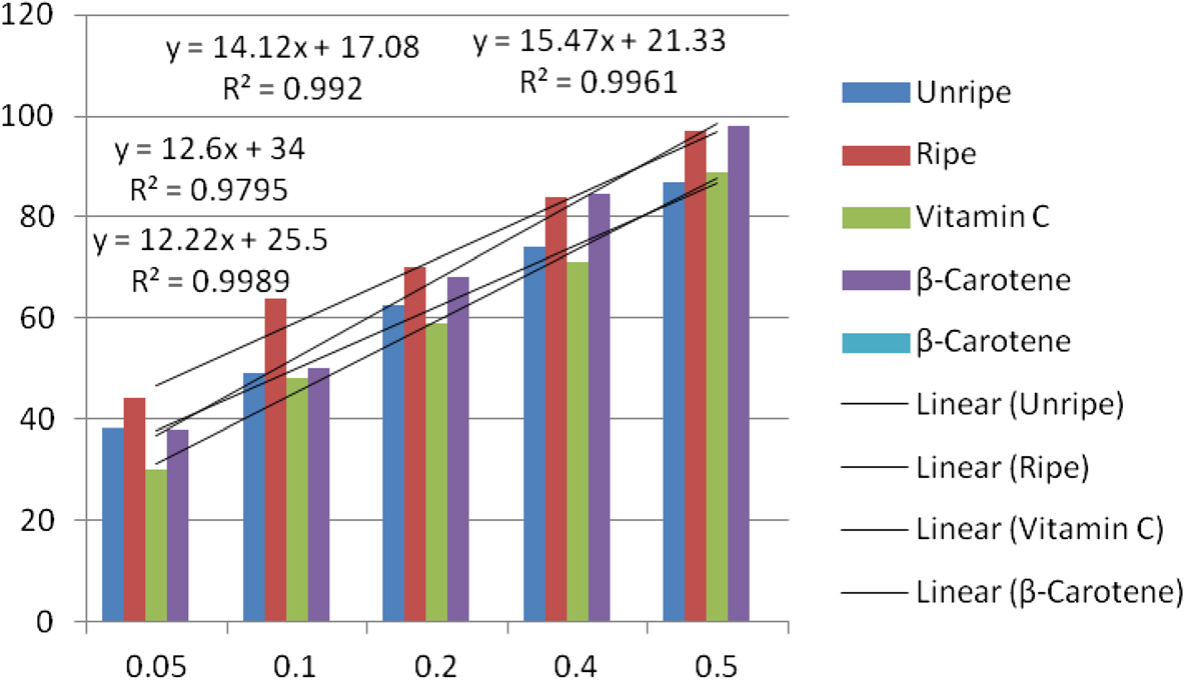
Antiradical activities of fruit volatile oil in *D. tripetala* and reference compounds on radicals of Nitric oxide

Antioxidant activities of UFO and RFO were examined using four (DPPH, ABTS, LP and NO) different radicals. The scavenging effects of the four free radicals by two oils and reference compounds were concentration dependant (Figs. 1, 2, 3 and 4). In the DPPH test (Fig. 1), the inhibitory effect of the two oils were stronger (#) than of reference compound (vitamin C) at all concentrations (0.05 to 0.50 mg/mL), however, when compared with β-carotene, results were similar (* *). The DPPH radical assay illustrates that a donor of electron or hydrogen atom is an antioxidant and its effect is demonstrated as DPPH•, color fades away (purple to yellow) in the test sample due to formation of neutral DPPH-H molecule upon absorption of hydrogen from an antioxidant [35]. The antioxidant strength of tested sample is evaluated by the decrease of UV absorption at 517 nm. However, DPPH test does not differentiate radical species but indicates general radical scavenging potential of an antioxidant [36]. Therefore, to evaluate specific antioxidant efficacy of UFO and RFO, quantitative tests using two different specific radicals species (LP • and NO•) and a cation (ABTS ^• +^) radical were carried out. A general trend in all four in vitro experiments was observed, where both the RFO and UFO of *D. tripetala* displayed valuable radicals scavenging effects acting as electron donors in the DPPH, ABTS tests, demonstrated high LP• as well as NO• radical scavenging capacity. Standard curves produced from % inhibitions against concentrations (Tables 2, 3, 4 and 5) of the oils and reference compounds (RC) were all linear in the four assays. Linear regression equation generated (Figs. 5, 6, 7 and 8) from each extract as well for the RC was used to calculate IC_50_ value. *T*-Test analysis was applied to test significant differences (Figs. 1, 2, 3 and 4) of % inhibitions against concentrations using SPSS15.0 for windows (IBM SPSS Inc *OLRAC SPS* registration number 2012/1786646/07). From the standard regression equations generated (Fig. 5) from DPPH data (Table 2), for UFO, y = 10.57× + 40.81; R^2^ = 0.9269: thus x (IC_50_ for UFO) = 50–40.81/10.57, = 0.87 mg/mL), for RFO, y = 13.49× + 41.59; R^2^ = 0.8214: × = 50–41.59/13.49, (IC_50_ for RFO) = 0.62 mg/mL, while for vitamin C, y = 14.512× + 0.87; R^2^ = 0.8978, × = 50–0.087/14.512 = 3.39 mg/mL and for β carotene, y = 11.64× + 46.312; R^2^ = 0.9139, × = 50–46.312/11.64, (IC_50_ for carotene = 0.32 mg/mL). The two oils reduced the DPPH• to a neutral DPPH-H, molecule, attaining 50% decrease with an IC_50_ value of 0.87 ± 0.23 mg/mL for the UFO, 0.62 ± 0.12 mg/mL for RFO, both lower than the value for vitamin C (3.39 ± 0.12 mg/mL) but significantly higher than that of β-carotene (0.32 ± 0.22 mg/mL) *p* < 0.05 (Table 2).

**Table 2:** Antioxidant capacity of fruits E. oils in *Dennettia tripetala* (mg/mL)

Activity	Essential oils *of D. tripetala*		Commercial Antioxidant (Positive Controls)	
	URO (IC_50_)	RFO (IC_50_)	Vitamin C (IC_50_)	β-Carotene (IC_50_)
DPPH ^•^	0.87 ± 0.23	0.62 ± 0.12	3.39 ± 0.12	0.32 ± 0.22
ABTS ^• +^	1.59 ± 0.11	0.90 ± 0.02	2.70 ± 0.03	0. 69 ± 0.13
LP ^•^	2.03 ± 0.10	1.90 ± 0.00	3.40 ± 0.10	1.67 ± 0.11
NO^•^	2.01 ± 0.12	1.27 ± 0.03	2.33 ± 0.11	1.85 ± 0.10

*URO* unripe fruit essential oil, *RFO* ripe fruit essential oil, ^• +^ cation radical, Values are mean ± SD, *n* = 3

The IC_50_ (mg/mL) was calculated from standard curve linear regression equation for each oil and positive controls

Significant difference was considered at a level of *P* < 0.05

In the ABTS test, the scavenging effects by the RFO and β-carotene were similar to that in DPPH model. However, unlike in the DPPH assay, UFO and vitamin C had low activity against ABTS ^• +^ at 0.05–0.2 mg/mL, while RFO and the reference compound (β-carotene) scavenging effects were moderate except at 0.4 and 0.5 mg/mL (Fig. 2). The % inhibitions against concentrations for ABTS data are presented Table 3, while the regression equations generated from the data (Fig. 6) are for UFO, y = 11.546× + 31.608; R^2^ = 0.9732. Thus x (IC_50_ for UFO) = 50–31.608/11.546 = 1.59 mg/mL; for RFO, y = 12.73× + 38.3, × (IC_50_ for RFO) = 50–38.3/12.73 = 0.92 mg/mL; for vitamin C, y = 10.638× + 21.072; R^2^ = 0.8935, × (IC_50_) = 50–21.072/10.638 = 2.70 mg/mL and β – carotene, y = 11.7× + 41.9; R^2^ = 0.9415, × = 50–41.9/11.7 = 0.69. The IC_50_ values obtained for RFO (0.90 ± 0.02 mg/mL) and β-carotene (0. 69 ± 0.13 mg/mL) from linear regression equations and *T*-Test analysis using SPSS15.0 for windows were higher compared to DPPH results. The discrepancy observed in the effects of the oils on DPPH and ABTS radicals might be due to factors including the ease at which the oils solvate the radical’s medium as well as complexity, polarity, preferred isomers selection of the radicals which are all factors suggested to influence the activity of volatile constituents in the scavenging of radicals [36].

**Table 3:** Percentage inhibitions of ABTS radical versus concentrations of EOs and Reference compounds

Conc.	%	Inhibitions		ABTS	radicals
mg/mL	Unripe	Ripe	Vit. C	β- carotene	
0.05	45	47.25	34	53	
0.10	55	60.2	44	61	
0.20	61	88	50	83	
0.40	80	92	55.48	92	
0.50	90.23	95	81.45	96	

% inhibitions are average of three parallel ABTS experiment on oils and reference compounds

The scavenging effects of UFO and RFO on lipid peroxide radicals (LP^•^) are showed in Fig. 3. The unripe and ripe fruit EOs exhibited more scavenging effect compared to the reference compound (vitamin C) at low concentrations (0.05 mg/mL). The UFO, RFO and β-carotene lipid peroxyl scavenging effects at low (0.05–0.10 mg/mL) and high (0.50 mg/mL) concentrations were similar (* *). At 0.2 and 0.40 mg/mL, β-carotene displayed more scavenging effect than the UFO and RFO (#), however at 0.5 mg/mL the two oils and β-carotene exhibited similar (**) scavenging activity. As in DPPH and ABTS assays, the average percentage inhibitions of lipid peroxyl radicals versus concentrations (Table 4) and the regression equations generated (Fig. 7) were used to calculate the IC_50_ values for EO and RC. The Interestingly, the IC_50_ values obtained for UFO and RFO (2.03 ± 0.10 and 1.90 ± 0.00 mg/mL respectively) were comparable to β-carotene (1.67 ± 0.11 mg/mL) and lower than that of vitamin C (3.40 ± 0.10 mg/mL) (Table 2). Fig. 4 shows the scavenging effects of UFO and RFO on nitric oxide radical (NO^•^) produced from red-colored complex salt of sodium nitroprusside solution at varying concentrations (0.05–0.5 mg/mL). Ripe fruit oil exhibited more scavenging activity on NO^•^ compared to UFO as well as the two reference compounds (β-carotene and vitamin C) at 0.05–0.20 mg/mL. At higher concentrations (0.4–0.5 mg/mL) the scavenging effects on NO^•^ by β-carotene and RFO were similar (**) and higher than that of UFO and vitamin C (Fig. 4). Overall, in the NO^•^ assay, the IC_50_ values obtained from standard curves regression equations generated (Fig.8) from the average % inhibitions in nitric oxide experiment (Table 5) for the two EOs and reference compounds indicated that RFO had the highest antioxidant capacity (1.27 ± 0.03 mg/mL), followed by β-carotene (1.85 ± 0.10 mg/mL), then UFO (2.01 ± 0.12 mg/mL) and vitamin C was least (2.33 ± 0.11 mg/mL) (Table 2).

**Table 4:** Percentage inhibitions of Lipid peroxidation radicals versus concentrations of EOs and reference compounds

Conc	(%	inhibitions	of	LP^a^	radicals
mg/mL	Unripe	Ripe	vit. C^b^	β-carotene	
0.05	40.3	42.45	21.23	38.01	
0.1	45.8	48.08	30.8	50.03	
0.2	58.4	60.3	33.07	77.02	
0.4	79.9	81.87	55.48	88.34	
0.5	93.4	95.4	81.45	91.00	

^a^lipid peroxyl radicals, ^b^vitamin C, (% inhibitions are average of three parallel LP experiment on oils and reference compounds

**Table 5:** Percentage inhibitions of nitric oxide radicals versus concentrations of EOs and reference compounds

Conc	%	inhibitions	of NO^a^	radicals
mg/mL	Unripe	Ripe	Vit.C^b^	β-Carotene
0.05	38.4	44.0	30.0	38.0
0.10	49.0	64.0	48.0	50.0
0.20	62.4	70.0	59.0	68.0
0.40	74.0	84.0	71.2	84.7
0.50	87.0	97.0	89.0	98.0

^a^Nitric oxide radicals, ^b^vitamin C (% inhibitions are average of three parallel NO experiment on oils and reference compounds)

The essential oils isolated from unripe and ripe fruits of *D. tripetala* displayed strong inhibitory effect against the 5 multi-drug resistant reference strains (*S. aureus* (NCINB 50080), *E. faecium* (ATCC19434), *L. ivanovii, (*ATCC 19119), *E. cloacae* (ATCC 13047), *E. coli* O157 (ATCC 700728), as well as 4 multi- drug resistant bacteria *E. coli* 180, *E. coli* 179*, E. coli* 132 and *Vibro* spp.) from our laboratory stock culture. The unripe fruit oil (UFO) was more active than the ripe fruit oil (RFO) against most of the bacterial strains investigated with MIC values of 0.05–0.20 mg/mL and 0.10–0.20 mg/mL respectively (Table 6). The UFO and RFO were bactericidal against *E. faecium* at 0.05 and 0.10 mg/mL respectively. The two essential oils were however bacteriostatic against the 8 multi-drug resistant bacterial strains after 24 h (Table 7). The two EOs showed lower activity against *E. coli* strains which are Gram negative bacteria when compared to Gram positive bacteria (*S. aureus, E. faecium, L. ivanovii, E. cloacae*) tested. The outer complex membrane of Gram-negative bacteria have been shown in previous studies to contain hydrophilic lipopolysaccharide [37], which create a barrier toward macromolecules and hydrophobic compounds, providing Gram-negative bacteria with higher tolerance toward hydrophobic antibacterial compounds like those found in essential oils [6, 7]. Another probable cause of resistance against phytochemicals has been suggested to be the presence of multi-drug resistant sites that promote the synthesis and secretion of amphipathic toxins [38]. The bioactivity of UFO and RFO tests on the multi-drug resistant bacteria also varied, the variation of components in their chemicals profiles (Table 1) may account for the observed results.

**Table 6:** Minimum Inhibitory Concentration (MIC) values (mg/mL) for E. oils of *D. tripetala*

Bacteria	UFO^a^	RFO^b^	Controls	
			Ciproflaxin Positive	DMSO negative
*Staphyloccocus aureus* (NCINB 50080)	0.10 ± 0.01	0.15 ± 0.01	0.05 ± 0.01	0.5 mL VG
*Enterococcus faecium* (ATCC19434)	0.05 ± 0.01	0.10 ± 0.02	0.05 ± 0.02	0.5 mL VG
*Escherichia coli (*ATCC 700728)	0.15 ± 0.02	0.20 ± 0.00	0.05 ± 0.02	0.5 mL VG
*Listeria ivanovii (*ATCC 19119)	0.10 ± 0.00	0.15 ± 0.01	0.05 ± 0.01	0.5 mL VG
*Enterobacter cloacae* (ATCC13047)	0.10 ± 0.01	0.10 ± 0.02	0.05 ± 0.02	0.5 mL VG
*Escherichia coli* 0179 (lab isolate)^c^	0.15 ± 0.02	0.20 ± 0.01	0.05 ± 0.02	0.5 mL VG
*Escherichia coli* 180 (lab isolate)^c^	0.20 ± 0.01	0.20 ± 0.00	0.05 ± 0.00	0.5 mL VG
*Vibro* spp. (lab isolate)^c^	0.05 ± 0.00	0.10 ± 0.01	0.05 ± 0.00	0.5 mL VG
*Escherichia coli* 132 (lab isolate)^c^	0.20 ± 0.00	0.20 ± 0.01	0.05 ± 0.02	0.5 mL VG

^a^:Unripe fruit oil, Ripe oil, ^b^:Ripe fruit oil, Ripe oil. *VG* visible growth, ^c^laboratory confirmed resistant isolates to

**Table 7:** Minimum Bactericidal Concentration (MBC) values (mg/ mL) for E. oils of *D. tripetala*

Bacteria	UFO ^a^	RFO ^b^	Ciproflaxin Positive control	DMSO Negative control
*Staphylococcus aureus* (NCINB 50080)	Bacteriostatic at 0.10 ± 0.01 VG	Bacteriostatic at 0.15 ±0.02 VG	Bactericidal at 0.05 ± 0.01 NVG	0.5 mL VG
*Enterococcus faecium* (ATCC19434)	Bactericidal at 0.05 ± 0.01 NVG	Bactericidal at 0.10 ± 0.02 NVG	Bactericidal at 0.05 ± 0.01 NVG	0.5 mL VG
*Escherichia coli* (ATCC 700728)	Bacteriostatic at 0.15 ± 0.02 VG	Bacteriostatic at 0.20 ± 0.00 VG	Bactericidal at 0.05 ± 0.01 NVG	0.5 mL VG
*Listeria ivanovii* (ATCC 19119)	Bacteriostatic at 0.10 VG	Bacteriostatic 0.15 VG	Bactericidal at 0.05 ± 0.01NVG	0.5 mL VG
*Enterobacter cloacae* (ATCC13047)	Bacteriostatic at 0.10 VG	Bacteriostatic at 0.10 VG	Bactericidal at 0.05 ± 0.01 NVG	0.5 mL VG
*Escherichia coli* 0179 *	Bacteriostatic at 0.15 VG	Bacteriostatic at 0.20 VG	Bactericidal at 0.05 ± 0.01 NVG	0.5 mL VG
*Escherichia coli* 180 *	Bacteriostatic at 0.20 VG	Bacteriostatic at 0.20 VG	Bactericidal at 0.05 ± 0.01 NVG	0.5 mL VG
*Vibro* spp. *	Bacteriostatic at 0.05 VG	Bacteriostatic at 0.10 VG	Bactericidal at 0.05 ± 0.01 NVG	0.5 mL VG
*Escherichia coli* 132*	Bacteriostatic at 0.20 VG	Bacteriostatic at 0.20 VG	Bactericidal at 0.05 ± 0.01 NVG	0.5 mL VG

^a^:Unripe fruit oil, Ripe oil, ^b^:Ripe fruit oil, Ripe oil. VG = visible growth, *laboratory confirmed resistant isolates to antibiotics

The bioactive compounds in EOs are broadly divided into four groups according to their chemical structure: terpenes, terpenoids, phenylpropenes, and others contain different degradation products originating from terpenes, unsaturated fatty acids, lactones, glycosides, and sulfur- or nitrogen-containing constituents. The constituents of the two EOs of *D. tripetala* in this current study were predominantly terpenoids. Monoterpenoids as well as sesquiterpenoids were more prominent (95.01%) in the profile of RFO than in UFO (Table 1). Previous essential oils studies have demonstrated bioactivities of some of the individual terpenes and terpenoids including p-cymene [6], α–terpinene, caryophyllene [39], linalool [40], β-ocimene [41] and ascorbic acid found in the EOs of *D. tripetala* in this present study. Several studies have indicated that *p*-cymene act as a substitutional impurity in the membrane, which partly perturbs the membrane of microorganisms [6]. Studies on cell and vesicle systems indicate that *p*-cymene has no effect on the membrane permeability, but acts by decreasing the enthalpy and melting temperature of membranes and also decreasing cell motility [42]. Alpha -terpinene found in both oils*,* had previously been reported by Takahashi et al. [43] which showed strong potency to inhibit low density lipoprotein oxidation even in the formation phase. Other components such as camphene, α – terpineol, α-pinene and borneol found in the two oils have been previously investigated and found to exhibit a variety of biochemical activities [44–46], these may have enhanced the activities of the EOs, suggesting possible synergistic interactions between the constituents. In addition, the main component- 2-methyl phenyl formate identified in the two oils for the first time could have reacted with the bacteria and other different radicals through various mechanisms as suggested by Mortein et al. [6] and Foti and Amorati [47]. The results from the current study are in agreement with other reports that have implicated aliphatic terpenes and terpenoids with bioactive properties, while the effect of cyclic monoterpenes and sesquiterpenes with double bonds were similar to the properties of phenolic compounds such as α – tocopherol. The ability of the UFO and RFO to scavenge four varieties of free radicals and exhibit strong activity against some multi-drug resistant bacteria is noteworthy. These observations may suggests that the fruit EO of *D. tripetala* could possibly be a new active candidate in the search for lead constituents for the management of infectious and oxidative stress-related disorders such as arthritis, cancers, arteriosclerosis, and dementia [48, 49].

## Conclusions

Present research indicates that in addition to local uses of the fruit, the essential oil in the fruit of *Dennettia tripetala* contained strong bioactive constituents and a potential candidate as new antimicrobial agent, as well as an alternative to synthetic antioxidant and may equally be used as preservatives in food.

## Abbreviations

ABTS^•+^: 2, 2-azino-bis (3-ethylbenzothiazolin - 6-sulfonic acid) diammonium salt radical
DMSO: Dimethyl sulphur oxide
DPPH^•^: 2, 2-diphenyl-1-picrylhydrazyl radical
GC-MS: Gas chromatography–mass spectrometry
IC_50_: Inhibitory concentration at 50%
LP^•^: Lipid peroxide radical
MBC: Minimum bactericidal concentration
MIC: Minimum inhibition concentration
NO^•^: Nitric oxide radical
RFO: Ripe fruit essential oil
UFO: Unripe fruit essential oil
